# Self-reported Nickel Allergy among Schoolchildren: Trends in Prevalence, Risk Factors, and Atopic Comorbidity

**DOI:** 10.2340/actadv.v105.42425

**Published:** 2025-04-14

**Authors:** Linnea HEDMAN, Malin LINDBERG, Berndt STENBERG, Eva RÖNMARK, Maja AF KLINTEBERG

**Affiliations:** 1 Department of Public Health and Clinical Medicine, The OLIN and Sunderby Research Unit, Umeå University, Umeå, Sweden; 2 Department of Public Health and Clinical Medicine, Dermatology and Venereology, Umeå University, Umeå, Sweden

**Keywords:** nickel, allergic contact dermatitis, ear piercing, atopic dermatitis

## Abstract

Nickel allergy is common among children. The present study investigated prevalence trends of self-reported nickel allergy, risk factors, and atopic comorbidity among children. Eight-year-old children from Norrbotten County, Sweden, were recruited in 1996 (*n* = 3,430), 2006 (*n* = 2,585), and 2017 (*n* = 2,785). Self-reported nickel allergy decreased from 7.7% (2006) to 6.1% (2017; *p =* 0.024) and was significantly more common among girls. In 1996, only children with atopic dermatitis answered questions on nickel allergy. Among children with atopic dermatitis, no significant decrease was seen over the years 1996 to 2017. Ear piercing (odds ratio [OR] 1.93, 95% confidence interval [CI] 1.39–2.68 and OR 5.57, 95% CI 3.71–8.38) and female sex (OR 4.05, 95% CI 2.68–6.13 and OR 1.73, 95% CI 1.09–2.74) were risk factors for self-reported nickel allergy in 2006 and 2017, respectively. Self-reported nickel allergy was significantly more prevalent among children with atopic dermatitis than without in 2006 (12.3% vs 6.4%; *p* < 0.001) and 2017 (11.5% vs 5.1%; *p* < 0.001), and among children with allergic rhinitis in 2017 (8.6% vs 4.7%; *p* = 0.015). In conclusion, we found a decreasing prevalence of self-reported nickel allergy, but not among children with atopic dermatitis. Ear piercing and female sex were strongly associated with nickel allergy. Our findings also suggest that nickel allergy is associated with atopic dermatitis and allergic rhinitis.

## SIGNIFICANCE

Nickel allergy is the most common contact allergy and studies on prevalence and risk factors are important in the preventive work of contact allergies. In the present study with 3 child cohorts included in 1996, 2006, and 2017, self-reported nickel allergy and risk factors were investigated. We found a decreased prevalence of self-reported nickel allergy, but not among children with atopic dermatitis. Self-reported nickel allergy was significantly more common among children with atopic dermatitis. We also found an association between self-reported nickel allergy and allergic rhinitis. Ear piercing and female sex were the strongest risk factors for self-reported nickel allergy.

Contact allergy (CA) is common in the general population, and nickel is the most common allergen causing allergic contact dermatitis, with a significant female predominance (1). In Europe, about 8–19% of adults and 8–10% of children and youths suffer from nickel allergy (NA) (1–4). NA is a cell-mediated hypersensitivity reaction caused by topical exposure to nickel. When the nickel ion penetrates the skin, it causes a proliferation of allergen-specific T-cells and memory T-cells. Renewed exposure to nickel activates the memory T-cells, subsequently causing the cellular damage and inflammation that characterize allergic contact dermatitis (5). Consumer goods capable of inducing NA are earrings, other jewellery, and metallic items on clothing and accessories, among others. Earrings are prominent risk factor for sensitization to nickel (4).

The European Nickel Directive came into force in 2001 to reduce the risk of developing NA. It was based on the Danish and Swedish national nickel regulations, implemented in 1990 and 1991, respectively. The directive limits the release rate of nickel for items that come into direct and prolonged contact with the skin. Since the introduction of the national nickel regulations and the European Nickel Directive, a decrease in prevalence of NA has been observed in several European countries (6). However, NA remains the most frequently occurring CA in the general population (1, 7, 8).

Patients with atopic dermatitis (AD) are more prone to react with skin irritation from different triggers. It has been proposed that AD could increase the risk of CA due to compromised integrity of the skin barrier (9). Also, patients with AD are more commonly exposed to possible allergens in emollients (10). Conversely, it has also been suggested that AD could be protective against CA because of differences in the immunological response of T helper cells (10). There are few studies on the relationship of NA with asthma and allergic rhinitis. A German study observed an association between NA and wheezing in both sexes, but only an increased risk of asthma among males (11). They could not find an association between rhinoconjunctivitis and NA. One previous study reported that nickel sensitization was less common in patients with allergic rhinitis compared with patients with non-allergic or overlapping rhinitis (12). Few studies have been conducted on the prevalence of NA and its trends in Northen Sweden, and on the potential association with ear piercing in this region. Epidemiological population-based studies are needed to investigate prevalence trends and risk factors for NA, and to clarify a possible association between NA and atopic diseases (7, 11–14).

This study aimed to determine the prevalence trends of NA among schoolchildren in Northern Sweden and its association with ear piercing. Given the implementation of the Nickel Directive, we hypothesize a declining trend. Other aims of this study were to investigate potential risk factors for NA and the association with atopic disease.

## Materials and methods

### Study area and population

The present study comprises 3 population-based paediatric cohorts within the research programme Obstructive Lung Disease in Northern Sweden (OLIN) studies. The recruitment years of the cohorts were 1996 (15), 2006 (16), and 2017 (17–19). All children in first and second grade from the municipalities of Piteå, Luleå, and Kiruna were invited to participate in a parental questionnaire survey. Piteå and Luleå are coastal towns, whereas the town of Kiruna is situated in the mountainous inland. All municipalities are in the county of Norrbotten, the northernmost county in Sweden. An identical study design was used for all 3 cohorts and has been described in previous publications (17–19). For each recruitment year, the median age of the participants was 8 years. The response rates were 97% (*n* = 3,430), 96% (*n* = 2,585) and 91% (*n* = 2,785) in 1996, 2006, and 2017, respectively.

### Questionnaire

The questionnaire comprised the validated International Study of Asthma and Allergy in Childhood (ISAAC) core questionnaire for asthma, rhinitis, and AD (20, 21). It also included additional questions concerning NA, ear piercing, atopic heredity, and environmental factors. In the cohort from 1996, only the children with AD answered the question regarding NA.

### Skin prick test

Children from Luleå and Kiruna were invited to participate in a skin prick test (17). Ten common airborne allergens were included. A mean wheal diameter ≥ 3 mm to at least 1 allergen was considered a positive reaction and defined as any allergic sensitization.

### Definitions

*Nickel allergy:* NA was defined by answering “yes” to the question “Has the child ever had symptoms of nickel allergy, i.e., itching/rash by jewellery, e.g., necklaces, earrings, metal buttons or buckles?”

*Atopic dermatitis:* AD was defined by affirmative answers to all 3 questions in the ISAAC questionnaire: “Has the child ever had an itchy rash which was coming and going for at least 6 months?”; “Has the child had this itchy rash at any time in the last 12 months?”; and “Has this itchy rash at any time affected any of the following places: the folds of the elbows, behind the knees, in front of the ankles, under the buttocks, or around the neck, ears or eyes?”

*Passive smoking in first year of life:* Mother or father smoking in the child’s first year of life.

*Asthma* and *allergic rhinitis* were defined as “any report of asthma” and “any report of allergic rhinitis” described in a previous publication (18).

### Statistical analysis

Statistical analyses were carried out using SPSS version 28.0 (IBM Corp, Armonk, NY, USA). To avoid overestimation of prevalence, internal missing (i.e. unanswered questions within a returned questionnaire) in questions on symptoms were considered negative responses. Internal missing in questions concerning exposures were regarded as missing and excluded from the analysis. The χ^2^ test was used for comparison of proportions between groups. A *p*-value < 0.05 was considered statistically significant. Logistic regression analysis was used to identify risk factors and protective factors for NA expressed as odds ratio (OR) with a 95% confidence interval (CI). Independent variables significantly associated with NA in the unadjusted analysis for each year were included in an adjusted logistic regression analysis for the corresponding year.

## Results

### Prevalence trends for nickel allergy

**Fig. 1:**
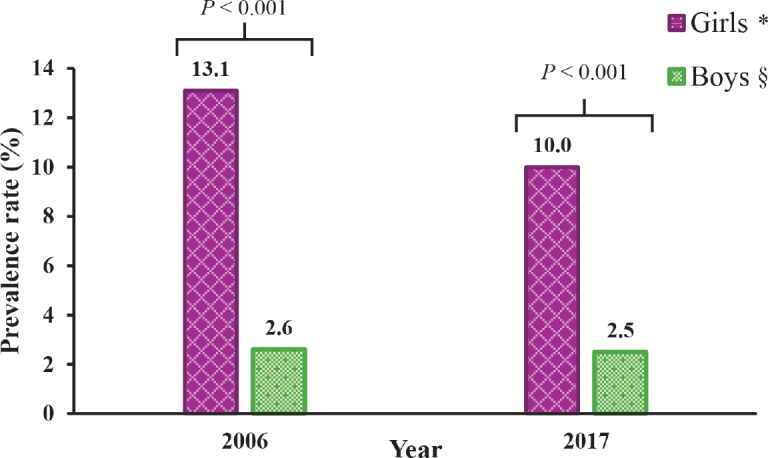
**Prevalence of self-reported nickel allergy among girls and boys in 2006 and 2017.** Differences by sex were analysed using the χ^2^ test. A *p*-value <0.05 was considered statistically significant. *Comparison among girls: 13.1% vs 10.0% (*p* = 0.012). §Comparison among boys: 2.6% vs 2.5% (*p* = 0.935).

The prevalence of self-reported NA among all schoolchildren decreased significantly from 7.7% (*n* = 198) in 2006 to 6.1% (*n* = 170) in 2017 (*p =* 0.024). When stratified by sex, the decreasing trend was significant only among girls, and girls had a significantly higher prevalence of self-reported NA in both 2006 and 2017 (**Fig. 1**).

**Fig. 2:**
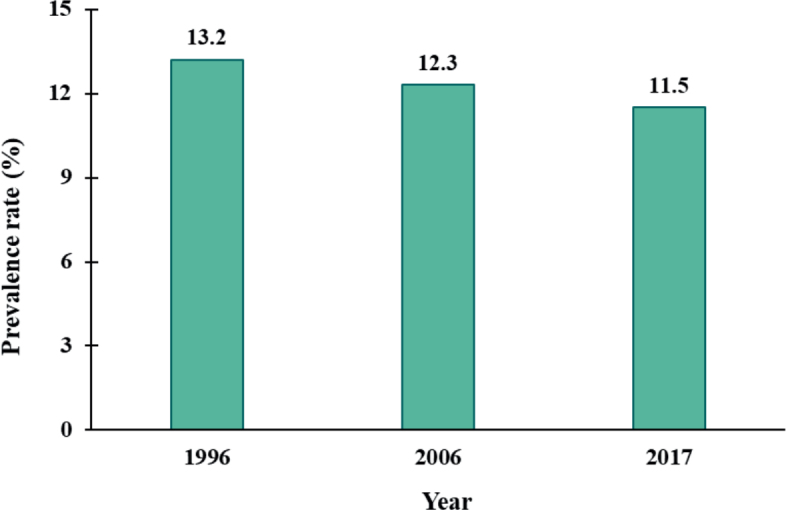
**Change in prevalence of self-reported nickel allergy among children with atopic dermatitis in 1996 (*n* = 781), 2006 (*n* = 551), and 2017 (*n* = 453).** Differences by year were analysed using the χ^2^ test, *p-*value = 0.676.

No significant changes in the prevalence of self-reported NA were found among children with AD between 1996, 2006, and 2017 (**Fig. 2**).

### Risk factors and protective factors for self-reported nickel allergy

A description of the study population and the prevalence of potential risk factors are presented in **Table I**. The prevalence of ear piercing increased significantly from 18.2% in 1996 to 25.6% in 2006, then plateaued in 2017 at 25.2% (*p* < 0.001). More girls than boys had pierced ears with a significant difference of 31.7% vs 5.3% in 1996 (*p <* 0.001), 46.7% vs 5.8% in 2006 (*p <* 0.001) and 47.2% vs 4.7% in 2017 (*p <* 0.001). Ear piercing was more common among children with self-reported NA than without. In 2006, 52.8% of children with self-reported NA had ear piercings compared with 23.4% without NA (*p* < 0.001). In 2017, 68.5% of children with self-reported NA had pierced ears vs 22.4% without NA (*p <* 0.001).

**Table I:** Description of the study population and prevalence of potential risk factors for self-reported nickel allergy in 1996, 2006, and 2017

Factor	1996 *n* = 3,430, % (*n*)	2006 *n* = 2,585, % (*n*)	2017 *n* = 2,785, % (*n*)	Difference by year, *p*-value
Female sex	49.0 (1681)	48.4 (1252)	48.3 (1346)	0.845
Atopic heredity	55.4 (1900)	58.2 (1505)	60.5 (1686)	**< 0.001**
Low birthweight (< 2,500 g)	4.2 (139)	3.7 (92)	5.0 (134)	0.058
Inland living	19.5 (670)	19.7 (509)	16.5 (460)	**0.003**
Heavy traffic road close to home	41.4 (1380)	41.9 (1055)	40.9 (1110)	0.750
House dampness	17.5 (586)	12.3 (309)	14.5 (393)	**< 0.001**
Passive smoking in first year of life	36.7 (1224)	19.6 (494)	11.9 (325)	**< 0.001**
Ear piercing	18.2 (618)	25.6 (655)	25.2 (692)	**< 0.001**
Indoor sports on a regular basis	62.6 (2133)	77.3 (1997)	76.1 (2120)	**< 0.001**
Ever dog	34.7 (1181)	36.1 (928)	38.7 (1066)	**0.004**
Daycare before the age of 2	58.5 (2005)	70.9 (1832)	80.5 (2243)	**< 0.001**
Living on or close to a farm	18.9 (626)	16.1 (404)	16.6 (453)	**0.010**
Outdoor sports on a regular basis	56.7 (1946)	61.0 (1576)	59.1 (1647)	**0.004**
Breastfeeding ≥6 months	56.0 (1922)	71.8 (1857)	68.5 (1898)	**< 0.001**
Older siblings	60.8 (2016)	62.6 (1565)	57.4 (1572)	**< 0.001**

Results obtained by χ^2^ test. A *p*-value < 0.05 was considered statistically significant and is highlighted in bold.

Factors included in the unadjusted and adjusted regression analyses for 2006 and 2017, along with their association with self-reported NA, are presented in **Table II**. Significant risk factors for NA in the unadjusted analysis for both years were female sex, passive smoking in first year of life, and ear piercing. Outdoor sports on a regular basis was consistently associated with a decreased risk in both cohorts and breastfeeding ≥ 6 months was associated with reduced odds in 2006 (Table II).

**Table II:** Factors associated with self-reported nickel allergy among children in 2006 and 2017

Factor		Unadjusted				Adjusted			

		2006		2017		2006		2017	

		OR	95% CI	OR	95% CI	OR	95% CI	OR	95% CI
Non-environmental risk									
Sex	Male	1		1		1		1	
	Female	**5.76**	3.95–8.41	**4.31**	2.96–6.28	**4.05**	2.68–6.13	**1.73**	1.09–2.74
Atopic heredity	No	1		1					
	Yes	1.31	0.97–1.77	1.09	0.79–1.49				
Low birth weight< 2500g	No	1		1					
	Yes	0.54	0.20–1.48	0.82	0.38–1.78				
Environmental risk factors									
Inland living	No	1		1					
	Yes	1.07	0.75–1.53	1.00	0.66–1.51				
Heavy traffic road close to home	No	1		1					
	Yes	0.89	0.66–1.20	1.20	0.87–1.65				
House dampness	No	1		1					
	Yes	1.06	0.68–1.64	1.46	0.98–2.18				
Passive smoking first year of life	No	1		1		1		1	
	Yes	**1.45**	1.03–2.03	**1.88**	1.26–2.82	1.29	0.90–1.85	1.33	0.86–2.05
Ear piercing	No	1		1		1		1	
	Yes	**3.67**	2.73–4.94	**7.52**	5.36–10.55	**1.93**	1.39–2.68	**5.57**	3.71–8.38
Indoor sports on a regular basis	No	1		1					
	Yes	1.22	0.85–1.75	1.06	0.73–1.53				
Ever dog	No	1		1					
	Yes	0.98	0.72–1.33	1.35	0.99–1.84				
Daycare before the age of two	No	1		1		1			
	Yes	**1.80**	1.25–2.59	1.05	0.70–1.55	**1.61**	1.10–2.34		
Living on or close to a farm	No	1		1					
	Yes	1.04	0.70–1.55	0.91	0.59–1.41				
Protective factors									
Outdoor sports on a regular basis	No	1		1		1		1	
	Yes	**0.62**	0.46–0.82	**0.71**	0.52–0.97	**0.70**	0.51–0.94	0.74	0.53–1.03
Breastfeeding ≥ 6 months	No	1		1		1			
	Yes	**0.73**	0.54–0.99	0.80	0.58–1.11	0.73	0.52–1.01		
Older siblings	No	1		1					
	Yes	0.86	0.64–1.16	0.88	0.64–1.21				

Results obtained by logistic regression analyses.

Identified significant factors in the unadjusted analysis for each year were included in the adjusted logistic regression analysis for the corresponding year.

OR: odds ratio; CI: confidence interval. Statistically significant values are in bold.

In the adjusted analysis, female sex remained a significant risk factor in 2006 (OR 4.05, 95% CI 2.68–6.13) and 2017 (OR 1.73, 95% CI 1.09–2.74). Ear piercing was significantly associated with self-reported NA in both years, with an OR of 1.93 (95% CI 1.39–2.68) in 2006 and 5.57 (95% CI 3.71–8.38) in 2017. Outdoor sports on a regular basis was a significant protective factor in 2006 but not in 2017 (Table II). Breastfeeding ≥ 6 months tended to be a significant protective factor in 2006 (OR 0.73, 95% CI 0.52–1.01).

### Association with atopic diseases

Children with AD had a significantly higher prevalence of self-reported NA compared with children without AD, 12.3% (*n* = 68) vs 6.4% (*n* = 130) in 2006 (*p <* 0.001) and 11.5% (*n* = 52) vs 5.1% (*n* = 118) in 2017 (*p* < 0.001). When children with AD were excluded from the analysis, a significantly increased prevalence of self-reported NA was observed among children with allergic rhinitis compared with children without allergic rhinitis in 2017 (**Table III**). No significant association was found between self-reported NA and asthma or allergic sensitization (Table III).

**Table III:** Comparison of the prevalence of self-reported nickel allergy (NA) among children with and without allergic rhinitis, asthma, and any allergic sensitization in 2006 (*n* = 2,034) and 2017 (*n* = 2,332)

Factor	Allergic rhinitis	No allergic rhinitis	*p-*value	Asthma	No asthma	*p-*value	Any allergic sensitization^a^	No allergic sensitization^a^	*p*-value
Reported NA, % (*n*)									
2006	6.1 (10)	6.4 (120)	0.889	6.7 (11)	6.4 (119)	0.863	7.3 (23)	7.0 (40)	0.898
2017	8.6 (18)	4.7 (100)	**0.015**	5.6 (18)	5.0 (100)	0.651	6.0 (23)	5.4 (58)	0.664

Children with atopic dermatitis (AD) were excluded from the analysis. Differences in proportions were analysed using the χ^2^ test. A *p*-value < 0.05 was considered statistically significant.

Statistically significant values are in bold.

a Allergic sensitization to any of the 10 tested allergens in the skin prick test. Among children without AD, *n* = 1,311 in 2006 and *n* = 1,447 in 2017 participated in skin prick tests.

## DISCUSSION

This population-based study of 8-year-old children in northern Sweden showed a decreasing trend in the prevalence of self-reported NA between 2006 and 2017. Female sex and ear piercing were the main risk factors for self-reported NA, whereas outdoor sports on a regular basis was protective. We also found that children with AD self-reported NA more frequently; the same was observed among children with allergic rhinitis in 2017.

Two studies in southern Sweden (8, 14) observed a decreasing trend in prevalence of NA verified by patch test in younger age groups, which is consistent with our study along with observations from other European countries (6). These findings are most likely an effect of the European Nickel Directive. When stratified by sex, the decreasing trend was significant only among girls, which also aligns with prior research using patch testing (14, 22). The low and stable prevalence among boys suggests exposure to nickel from sources not covered by the Nickel Directive. These may include keys, paintbrushes, handbags, and laptops (23). Another potential source is mobile phones. Even though nickel release from mobile phones has been regulated since 2009, studies have shown that excessive nickel release still occurs frequently (23, 24).

We found a significant female predominance of self-reported NA and female sex was a significant risk factor, a well-known phenomenon reported previously in many different studies (4, 8, 14, 25–27). The female predominance is likely due to girls being exposed to nickel to a greater extent than boys. We observed a substantially higher frequency of ear piercing among girls, confirming one difference in exposure. However, also after adjusting for ear piercing, female sex remained as a risk factor.

In our study, children with self-reported NA had an increased prevalence of ear piercing compared with children without NA, in line with another Swedish study (28). It has also been shown that the prevalence of NA increased with the number of piercings (4). We observed that ear piercing was associated with elevated odds for NA both in 2006 and 2017, but with higher odds in 2017. It is possible that the increase could be caused by a rise in popularity of online shopping. Earrings ordered online may be imported from countries outside of the European Union, in which case the release rate of nickel could exceed the threshold specified in the Nickel Directive. It has been shown that 31.5% of earrings from China and 29.2% of Thai earrings exceeded the limit of nickel release in 2010 (29). Earrings sold within the European Union might also release excessive nickel, given that 14.8% of inexpensive earrings sold in Denmark in 2009 and 14.4% of post-assemblies sold on German markets in 2008 surpassed the limit (30, 31).

Previous studies have observed a potential association between tobacco smoking and NA in the adult population (32). We found a significant association between passive smoking in first year of life and elevated odds for NA in the unadjusted analysis but not in the adjusted analysis, indicating smoking only to be a marker for nickel exposure.

Children who attended daycare before the age of 2 had a significantly increased risk of NA in 2006. This could theoretically be explained by an earlier exposure to nickel from toys, pens, paintbrushes, and other metal-containing items found at daycare. One study from 2017 found that nickel was deposited on the skin after short-term play with 2 out of the 3 tested children’s toys (33). It is possible that toys contained more nickel when the children from the 2006 survey attended daycare, compared with the children from the 2017 cohort.

Outdoor sports on a regular basis was a significant protective factor for NA in 2006 and tended to be significant in 2017 as well. To our knowledge, no previous studies have reported such findings. It is possible that children regularly practising outdoor sports come into less contact with metal-containing equipment, toys, or accessories. However, the results should be interpreted with caution as the question did not specify which sports the children were practising. Hence, further studies are needed, preferably qualitative, where the participant’s life habits and exposures can be explored.

In our study we investigated whether breastfeeding could protect against NA. In the unadjusted analysis, we found a slightly protective effect in 2006, but only a tendency when adjusted for other risk factors. Oral exposure to nickel, with nickel-containing braces, has previously been shown to be protective against NA (34). There might be difference in oral nickel exposure between breastfed children and children fed with infant formula. However, our study did not analyse levels of nickel in breastmilk and infant formula.

In our study we found no significant change in self-reported NA among children with AD. However, compared with children without AD, those with AD had a significantly higher prevalence of self-reported NA. One explanation could be that individuals with AD have an impaired epithelial barrier with increased permeability, making them more vulnerable to penetration by allergens (9). However, atopic skin is more prone to react with irritative dermatitis when in contact with metals, which might be misinterpreted as allergic dermatitis caused by nickel (35). It is possible that irritant reactions among children with AD are misinterpreted as being NA. Such misinterpretations may lead to an overestimation of the true prevalence, possibly explaining the lack of a declining trend of NA among children with AD in our study (36). Previous studies have shown contradictory results on the association between CA and AD and in a systematic review no association was found between AD and patch test verified contact sensitization (37). Previous studies have also observed a higher prevalence of AD and increased likelihood of irritative reactions to metals in cold and dry climates (18, 38). The winter season in northernmost Sweden offers a dry and cold climate, with lower temperatures and humidity levels inland than along the coast. However, living in the mountainous inland was not a significant risk factor for self-reported NA in our study. Future studies investigating potential variations in the prevalence of nickel sensitization across different climate zones are warranted.

To our knowledge, we are the first to observe a positive association between self-reported NA and allergic rhinitis. It might be that children with allergic rhinitis, despite no AD, have more sensitive skin, reacting with irritation to triggers, and this needs to be further studied preferably with patch testing. One study observed a higher frequency of metal sensitization in individuals with other types of rhinitis than allergic (12), while another (39) did not find an association between CA and AD or inhalant allergy. Consistent with this study and another, we did not find an association between NA and asthma (13). Conversely, one study reported a higher prevalence of NA in asthmatic patients compared with non-asthmatic patients, and another study found that reported NA increased the risk for asthma among males (11, 40). The prevalence of airborne allergen sensitization in our study population increased between 1996 and 2006, then stabilized and remained unchanged at 30% in 2017 (17). Despite IgE-mediated sensitization being prevalent, we did not find it associated with self-reported NA.

One limitation of our study was the lack of data on the prevalence of self-reported NA in 1996, when only children with AD answered the question regarding NA. Limitations of self-reported data are the risk of potential recall and reporting bias, and that the self-reported prevalence of NA may overestimate the actual occurrence (2, 36). However, our prevalence estimate aligns with previous findings among Swedish adolescents (2, 3). Another limitation of our study is that the self-reported NA was not validated by a patch test. A Swedish study among youths and young adults comparing questionnaire data and patch tests (2) found a prevalence of NA of 15% based on self-reports and 10% based on patch testing. If the ratio between self-reported NA and positive patch tests for nickel had been equivalent in our study population, the true prevalence would have been around 4–5%. This could indicate a lower prevalence of NA among children in Norrbotten compared with southern regions. However, it could also be due to the young age of the participants in our study. The major strengths of our study were the high number of participants and the high response rates, which reduce the risk of selection bias and strengthen the reliability of the estimated prevalences. Other strengths are that the surveys were identical in terms of the study population’s age, the geographical area, and the methods used. In clinical practice, the most commonly used diagnostic criteria for AD are the UK working party criteria; however, in larger epidemiological studies the ISAAC questionnaire is widely used, has been well validated over time, and has shown sufficient accuracy when estimating prevalence on a population level (20, 21). The ISAAC questionnaire is based on symptoms in the last 12 months, which reduces the risk of recall bias and reflects a current disease burden, in contrast to lifetime prevalence, which can overestimate by including cases no longer clinically relevant.

In conclusion, our results indicate a decreasing trend in prevalence of self-reported NA among schoolchildren in Norrbotten County. Female sex and ear piercing were significant risk factors for self-reported NA. Outdoor sports was found to be a significant protective factor. Our findings also suggest an increased comorbidity between self-reported NA and AD and between self-reported NA and allergic rhinitis. The association with allergic rhinitis calls for further studies, as does our finding of a negative association between outdoor sports and self-reported NA.
